# Alpinetin inhibits breast cancer growth by ROS/NF‐κB/HIF‐1α axis

**DOI:** 10.1111/jcmm.15371

**Published:** 2020-06-20

**Authors:** Tao Zhang, Shuai Guo, Xinying Zhu, Jinxia Qiu, Ganzhen Deng, Changwei Qiu

**Affiliations:** ^1^ Department of Clinical Veterinary Medicine College of Veterinary Medicine Huazhong Agricultural University Wuhan China

**Keywords:** alpinetin, apoptosis, breast cancer, HIF‐1α, NF‐κB, ROS

## Abstract

Alpinetin, the main active ingredient in the Chinese medicinal herb *Alpinia katsumadai Hayata*, has been found to have anticancer activity. However, the therapeutic efficacy of signalling cascades modulated by alpinetin remains unknown. Here, we showed that alpinetin provoked mitochondria‐associated apoptosis in a dose‐dependent manner in breast cancer cells. Mechanistic investigations revealed that alpinetin dampens hypoxia‐inducible factor‐1α (HIF‐1α) signalling due to a lack of NF‐κB activation through reduced mitochondrial reactive oxygen species (ROS) production, decreasing HIF‐1α transcription. In vivo, we also found alpinetin led to significant tumour regression by inhibiting NF‐κB pathway. Overall, our work uncovers a ROS/NF‐κB/HIF‐1α axis‐dependent mechanism underlying the anticancer effects of alpinetin and suggests that alpinetin could act as a novel therapeutic agent against breast cancer.

## 1 INTRODUCTION

As expected, cancer is the leading cause of death and the single most important obstacle to increase life expectancy in every country in the world in the 21st century.^1^ Chemotherapy is one of the effective treatments for metastatic cancers. However, serious side effects of chemotherapy drugs that kill normal cells and cancer cells without discrimination and the ability of cancer cells to evade apoptosis have remained significant impediments to successful chemotherapy.^2^ For example, vinorelbine, paclitaxel and anthracyclines were found to be associated with multidrug resistance.^3^, ^4^ Therefore, revealing the anticancer mechanisms and screening novel chemotherapy drugs to circumvent drug resistance are likely to improve chemotherapy.

Alpinetin, a natural flavonoid, is the major active constituent of the traditional medicinal plant *Alpinia katsumadai Hayata,* which has been used medicinally since ancient times.^5^, ^6^ Alpinetin has been reported to have anticancer activity, including apoptosis induction, cell cycle arrest and proliferative suppression in many types of cancer, such as breast cancer, lung cancer, colon cancer and liver cancer.^7^, ^8^, ^9^, ^10^ In addition to anticancer activity, alpinetin has shown considerable anti‐inflammatory activities. Alpinetin has been reported to inhibit NF‐κB activation and decrease oxidative stress to suppress the release of proinflammatory cytokines, such as tumour necrosis factor (TNF)‐α and interleukin (IL)‐1.^11^, ^12^ However, the imprecise mechanisms of action and its unknown direct target have greatly hindered its clinical application in the treatment of cancer.

Mitochondria are the vital of energy metabolism that produces ATP through oxidative phosphorylation and acts as centre cellular signalling hubs.^13^, ^14^ It is generally believed that tumour cells are in a relatively hypoxic cellular environment in solid tumours, and energy harvesting is dependent on the glycolytic pathway.^15^ Under conditions of low oxygen tension, the cellular metabolism, function and fate that is governed by altered gene expression initiated by the transcription factor HIF‐1.^16^ Therefore, HIF‐1 was found to be highly expressed in a variety of cancers, thereby adapting to the hypoxic environment in the cancer microenvironment and avoiding cancer cell death.^17^ In addition, mitochondria involved in the production of reactive oxygen species (ROS) that cause severe cellular damage. Studies have shown that ROS is involved in various biological processes of cancer cell development.^18^, ^19^ Thus, inhibiting the expression of HIF‐1 and blocking ROS generation may be a potential target for cancer treatment.

In this present study, we examined the effect of alpinetin in three breast cancer cell lines and investigated the mechanisms by which alpinetin regulates HIF‐1α expression and induces cancer cell death. Data demonstrate that alpinetin dose‐dependently inhibit NF‐κB pathway activity by decreasing cellular ROS production, thereby inhibiting HIF‐1 expression.

## 2 METHODS

### 2.1 Reagents, antibodies and general methods

**FIGURE 1:**
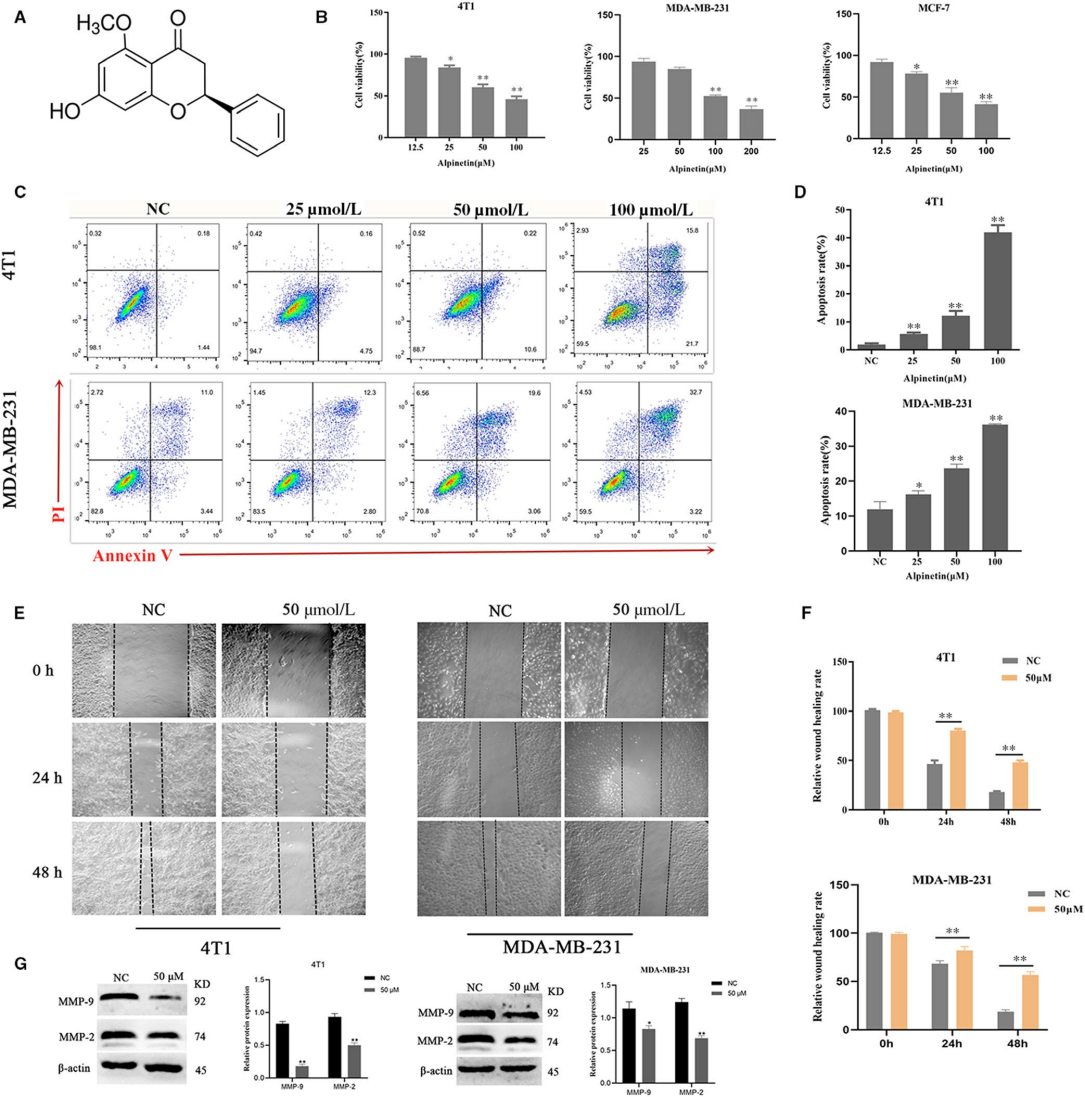
Alpinetin exerts significant cytotoxicity in breast cancer cells. A, Chemical structure of alpinetin. B, CCK‐8 kits were used to assess the proliferation of 4T1, MCF‐7 and MDA‐MB‐231 cells treated with alpinetin for 24 h. C and D, Cell apoptosis assays of 4T1 and MDA‐MB‐231 cells treated with alpinetin (25 μmol/L, 50 μmol/L and 50 μmol/L) using flow cytometry. Cells were collected and labelled with Annexin V‐FITC and PI. E and F, Wound‐healing assays of 4T1 and MDA‐MB‐231cells after alpinetin (50 μmol/L) treatment. Representative images depicting the *t* = 0 h, *t* = 24 h and *t* = 48 h time‐points of the recording period are shown. G, Western blot analysis of MMP‐9 and MMP‐2 after 4T1 (left) and MDA‐MB‐231(right) cells were treated with alpinetin (50 μmol/L) for 24 h. β‐Actin was used as an internal control. All results are expressed as the mean ± SEM of three independent experiments. The symbols * and ** denote significant differences of *P* < .05 and *P* < .01, respectively

Alpinetin (Figure 1A, molecular formula C_16_H_14_O_4_, molecular weight 270.28, purity ≥ 98%) purchased from Yuanye Bio‐Technology Co., Ltd. Dimethyl sulfoxide (DMSO) was purchased from Sigma. Alpinetin dissolved in DMSO at a concentration of 25, 50 and 100 mmol/L before use in vitro experiments. The final concentration of DMSO was kept below 0.1% in all cell cultures. When used in mice, alpinetin was dissolved in DMSO at a stock concentration of 100 mg/mL and freshly diluted with 0.09% NaCl. Primary antibodies for β‐actin (#3700) and anti‐p‐NF‐κB p65 (#3033), anti‐NF‐κB p65 (#8242), anti‐p‐IκBα (#2859) and anti‐IκBα (#9242) were purchased from Cell Signaling Technology. Anti‐cleaved caspase‐3 (#ab184787), anti‐cleaved caspase‐9 (#ab2324), anti‐cleaved PARP (#ab32064) and anti‐HIF‐1α (#ab179483) were obtained from Abcam. Anti‐Bax (#sc‐493) and anti‐Bcl‐2 (#sc‐492) were obtained from Santa Cruz Biotechnology. Anti‐Cytochrome c (#GB11080), anti‐COX IV (#GB11250) and ki67 (#GB13030‐2) were purchased from Servicebio.

### 2.2 Cells and cell culture conditions

4T1 cells were purchased from the Chinese Academy of Sciences cell Bank. MCF‐7 and MDA‐MB‐231 were purchased from the American Tissue Culture Collection (ATCC). Cells cultured under 5% CO_2_ and ambient O_2_ at 37°C in RPMI 1640 (Invitrogen) containing 10% foetal bovine serum (FBS, PAN), 100 IU/mL penicillin and 100 μg/mL streptomycin (HyClone).

### 2.3 Mice and in vivo studies

Eight‐week‐old BALB/C female mice were purchased from the Hubei Provincial Center for Experimental Animal Research. Mice were maintained under free diet condition at the room with temperature controlled of 25°C. The animal protocol was approved by the Institutional Ethical Committee for Animal Care and Use of Huazhong Agricultural University and was in line with the United States National Institutes of Health's published experimental animal care and use guide (HZAUMO‐2015‐12). There were approximately 1.0 × 10^7^ MDA‐MB‐231 cells without any contamination that were harvested, suspended in 100 μL of PBS and then subcutaneously injected into each mouse's fourth breast pad. Obvious lumps were visible 1 week later. Mice were randomly two groups, six mice in the control group and nine mice in alpinetin group, as follows: the control group (20 μL DMSO i.p. three times a week); the alpinetin group (100 mg/kg 20 μL alpinetin i.p. three times a week). The tumour size and weight of the mice were measured every 2 days. Tumour volume (V) was calculated according to the formula V = 0.5 × L × W^2^, where L is the greatest diameter and W is the diameter at the point perpendicular to L. One month later, mice were killed and the tumour was collected to next experiment.

### 2.4 Cell transfection

Transient transfection of 4T1 cells was carried out using Lipofectamine 2000 (Invitrogen) as recommended by the manufacturer. HIF‐1α siRNA, overexpression pcDNA3.1 + HIF‐1α vector and their respective negative control oligonucleotides were purchased from GenePharma.

### 2.5 Cell viability wound‐healing and apoptosis assays

The cells (5 × 10^3^ cells/mL) were seeded onto 96‐well plates at 37°C for 12 hours, with five parallel wells in each group. The effect of drugs on the number of viable cells was evaluated using CCK‐8 (Dojindo Laboratories) assays according to the manufacturer's instructions. Cell viability = (Treatment Group OD−Blank Group OD)/(Control Group OD−Blank Group OD) × 100%.

A wound‐healing assay was used to assess cell migration. As a previously described method in a study,^20^ the cells were seeded onto 6‐well plates. After cells were almost covered, wounding was accomplished by dragging a 200 μL pipette tip through the monolayer and then washing the cells twice. The 4T1 and MDA‐MB‐231 cells culture with serum‐reduced Opti‐MEM I medium (Invitrogen), and the wound closures were photographed when the scrape wound was introduced (0 hour) and at a designated time (24 and 48 hours) using an inverted microscope.

The flow cytometry analysis was used to measure cell apoptosis. Stained cells were analysed using the Annexin V‐PI apoptosis detection kit (BD) according to the manufacturer's protocol and were analysed by FACS (BD).

### 2.6 Quantitative RT–Qpcr

Total RNA was isolated using the TRIzol^®^ reagent (Invitrogen), and cDNA was synthesized using the HiScript^®^ II Q Select RT SuperMix for qPCR kit (Vazyme Biotech Co., Ltd). Quantitative RT‐qPCR was performed in duplicates using FastStart Universal SYBR Green Master (Roche Applied Science) using the StepOne real‐time PCR System (Life Technologies Corp.). GAPDH was used as an endogenous normalization control to obtained relative expression data. The expression levels of miRNA were assessed using a Hairpin‐it™ microRNA qPCR Quantitation Kit (GenePharma) according to the standard protocol. The expression of U6 was used as an endogenous control. All primer sequences are shown in Table 1.

**TABLE 1:** Primer sequence for qPCR

Gene	Primer sequence (5′‐3′)	product size (bp)
HIF‐1α	Forward: GGAAGCCGGTTCCTGAGAG	115
	Reverse: GGCCGGACTTTCTCCTGTTC	
GAPDH	Forward: AACAGCAACTCCCACTCTTC	111
	Reverse: CCTGTTGCTGTAGCCGTATT	
GLUTI	Forward: GGGAGCTAACATCTCCAAGTCT	167
	Reverse: CTGGCATCAACGCTGTCTTC	
PDK1	Forward: CTTGAATTCGAGTGCGGAGA	252
	Reverse: GCCGGCCAAGACTCGAGTTT	

### 2.7 Western blot analysis

Cell or tissue were lysed in RIPA solution involving a phosphatase inhibitor (Vazyme), immobilized protein on beads in sample reducing buffer followed by denaturing at 95°C for 5 minutes. The protein concentrations were determined using the BCA protein assay kit (Vazyme). The total protein was separated by SDS‐PAGE and transferred onto PVDF membranes. Blots were successive incubated with antibodies and secondary antibodies. Anti‐β‐actin was used as control. Protein expression was detected using an Enhanced Chemiluminescence Detection System (ImageQuant LAS 4000 mini).

### 2.8 Measurement of ROS production

The intracellular ROS level was measured by flow cytometry. Briefly, cells were seeded at a density of 1 × 10^6^ cells/mL into 6‐well plates and, after treatment as indicated, the intracellular ROS level was measured using the oxidative conversion of cell permeable 2′,7′dichlorofluorescein diacetate (DCFH‐DA, Beyotime) to fluorescent dichlorofluorescein (DCF). The cells were incubated with DCFH‐DA for 30 minutes at 37°C, then washed again, and flow cytometry was performed.

### 2.9 Immunofluorescence staining

Cells with or without alpinetin treatment were collected and fixed with 4% paraformaldehyde for 10 minutes, loaded onto coverslips and dried, and permeabilized with 0.2% Triton X‐100 for 10 minutes before they were blocked with 5% BSA for 1 hour. Cells were incubated with an antibody overnight at 4°C and then incubated with secondary antibody in the dark for 2 hours at 25°C. After incubating cells with DAPI (5 mg/mL) for 10 minutes (DAPI, Beyotime) and observed using fluorescence microscopy (Olympus). PBS was used for all washing steps.

### 2.10 Histological analyses and immunohistochemistry

Tissues were fixed in 4% formaldehyde solution, embedded in paraffin, and sections were stained with haematoxylin and eosin (H&E). Immunohistochemistry detection using anti‐Ki67 was performed on paraffin sections. The staining processes were performed according to standard methods. The sections were observed using an optical microscope (Olympus).

### 2.11 TUNEL assay

TdT‐UTP nick‐end labelling (TUNEL) assay was performed a TUNEL assay kit (Roche Diagnostics GmbH) according to the manufacturer's instructions as described previously.^21^

### 2.12 Statistical analysis

The values are expressed as mean ± SEM and *P* values were calculated as detailed in the corresponding legends. The unpaired *t* test (GraphPad Software) was used for statistical analysis and to generate graphs. Replicates are biological replicates. Student's *t* test was used to assess statistical significance (^∗^ *P* < .05, ^∗∗^ *P* < .01).

## 3 RESULTS

### 3.1 Breast cancer cells are sensitive to alpinetin

We first assessed the efficacy of alpinetin in 4T1, MCF‐7 and MDA‐MB‐231 cell lines by CCK‐8 assays. After 24 hours of treatment, the percentage of viable breast cancer cells was significantly reduced by alpinetin in a concentration‐dependent manner (Figure 1B). The results showed that the toxic effects of alpinetin on the three cells were substantially the same, so the next experiment was performed on both 4T1 and MDA‐MB‐231 cell lines. We next examined the effect of alpinetin on apoptosis. Flow cytometry analysis showed that alpinetin induced obvious apoptosis in a concentration‐dependent manner in 4T1 and MDA‐MB‐231 cells (Figure 1C,D). Similarly, the wound‐scraping assay indicated that alpinetin inhibited cell migration compared with DMSO treatment (Figure 1E,F). These results demonstrate that alpinetin is cytotoxic to breast cancer cells.

### 3.2 Alpinetin induces mitochondria‐associated apoptosis in breast cancer cells

**FIGURE 2:**
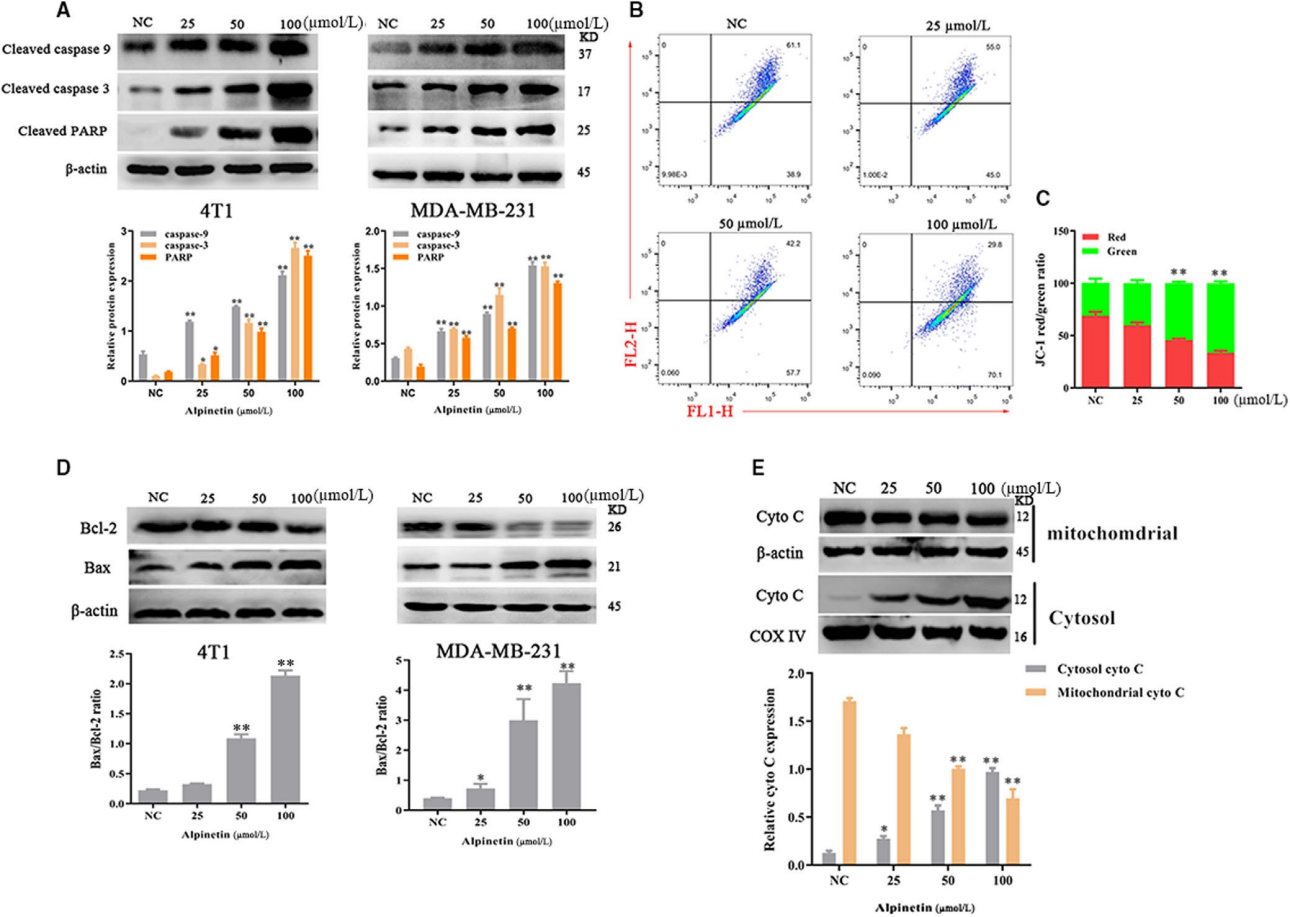
Alpinetin induces mitochondria‐associated apoptosis. A, 4T1 (left) and MDA‐MB‐231(right) cells were treated with the indicated doses of alpinetin for 24 h. Western blot analysis of the apoptotic proteins PARP, caspase‐9 and caspase‐3 in 4T1 cells. β‐Actin was used as an internal control. B and C, JC‐1 staining and flow cytometry were performed to evaluate the effect of alpinetin on MMP. The percentage of JC‐1 positive and JC‐1 negative cells for 4T1 cells is indicated in the left panel. D, Western blot analyses of Bax and Bcl‐2 after 4T1 (left) and MDA‐MB‐231(right) cells treatment with alpinetin for 24 h. β‐Actin was used as an internal control. E, Western blot analyses of cytochrome c after treatment with alpinetin for 24 h. β‐Actin was used as an internal control for cytoplasmic proteins. COX IV was used as an internal control for mitochondrial proteins. Data represent three independent experiments and are presented as the mean ± SE M (error bars). Two‐tailed Student's *t* test, *P < .05; **P < .01

To observe whether the apoptotic effect of alpinetin was activated by a cascade of caspases, the cleavage of cleaved caspase‐9/caspase‐3/PARP was detected by Western blot. As shown in Figure 2A, alpinetin caused activation of caspase‐9, caspase‐3, and PARP in 4T1 and MDA‐MB‐231 cells. Apoptosis consists of two classic pathways: the death receptor ‘extrinsic’ and mitochondrial ‘intrinsic’ pathways.^22^ Apoptosis induced by activation of the caspase‐9/3 cascade is suggested to involve the mitochondrial apoptotic pathway. Therefore, we determined the mitochondrial membrane potential (Δψm) via the JC‐1 fluorescent probe. As shown in Figure 2B,C, we found that alpinetin induced mitochondrial depolarization in 4T1 cells. Moreover, we examined the change in levels of Bax family proteins, which are key proteins in mitochondrial apoptosis, upon treatment of breast cancer cells with alpinetin and examined the release of cytochrome c (cyto‐c) from the mitochondria by Western blot. The results showed that the rate of Bax/Bcl‐2 release was remarkably elevated by alpinetin, and treatment with alpinetin induced cyto‐c release from mitochondria to the cytoplasm (Figure 2D,E). Altogether, these data suggest that the cellular uptake of alpinetin induces mitochondria‐associated apoptosis.

### 3.3 Alpinetin reduced ROS generation and then inhibited NF‐κB in breast cancer cell

**FIGURE 3:**
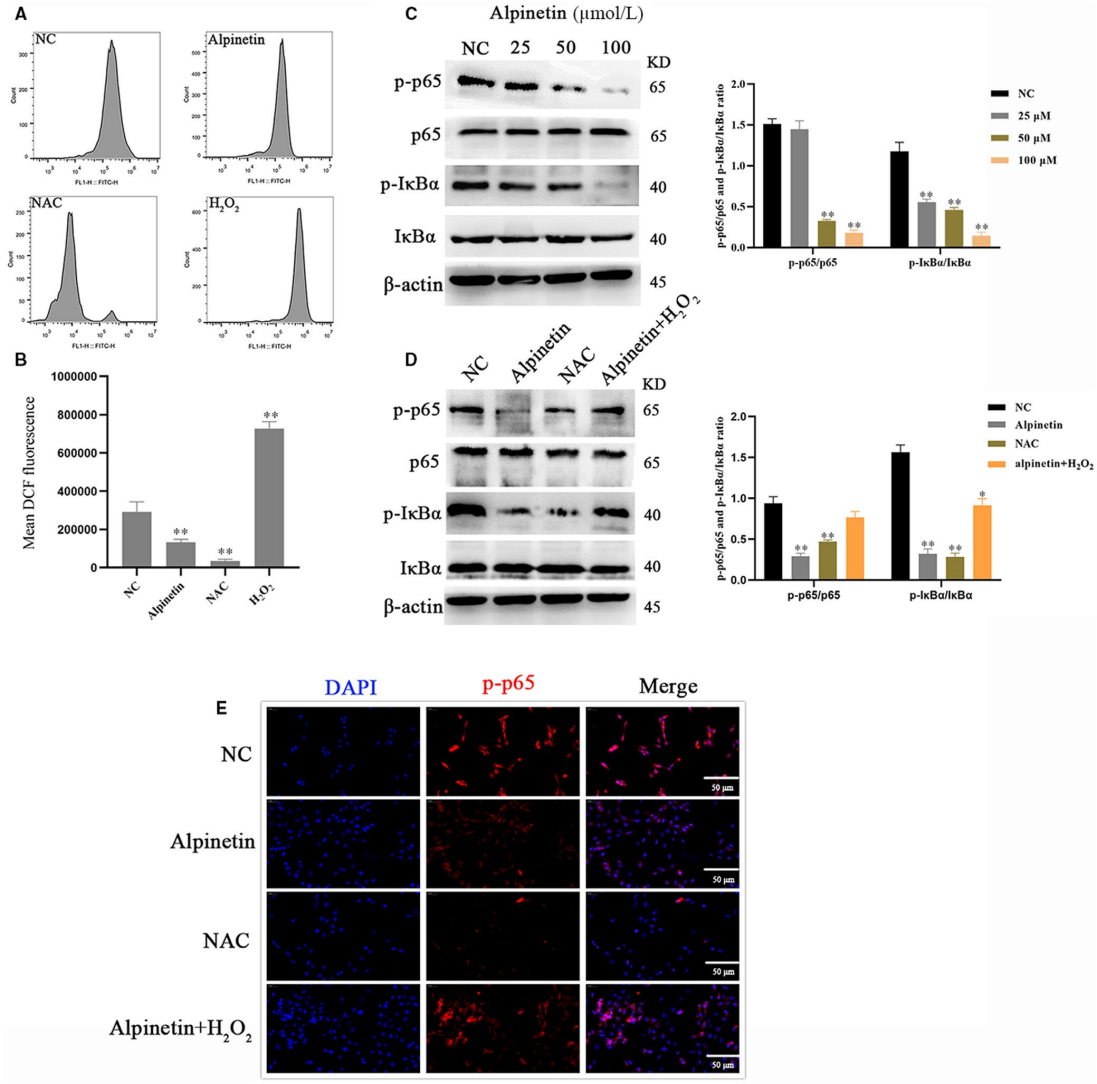
Alpinetin reduced ROS generation and then inhibited NF‐κB in breast cancer cell. A and B, ROS levels in 4T1 cells treatment with NAC or alpinetin or H_2_O_2_ for 24 h were assessed using flow cytometry. NAC (1 mmol/L): negative control; H_2_O_2_ (10 μmol/L): positive control. C and D, Western blot analysis of NF‐κB pathway key proteins levels under alpinetin in the presence and absence of 1 mmol/L NAC or 10 μmol/L H_2_O_2_ for 24 h using anti‐β‐Actin antibody as a control. E, Determination of NF‐κB nuclear translocation under alpinetin for 24 h in 4T1 cells by immunostaining the cells with antibodies against p‐p65 (red) and DAPI (blue). Data represent three independent experiments and are presented as the mean ± SE M (error bars). Two‐tailed Student's *t* test, *P < .05; **P < .01

Mitochondria are important sites for intracellular ROS production. Thus, the level of ROS in cancer cells was evaluated upon alpinetin treatment. Our results showed that alpinetin reduces intracellular ROS production (Figure 3A,B). Several studies have suggested that ROS is the key stimuli regulator of NF‐κB signalling,^23^ so we assay the activation of NF‐κB pathway. We found that alpinetin inhibits NF‐κB pathway activation in a dose‐dependent manner (Figure 3C) and increases ROS level to abolish the effect of alpinetin in inhibiting NF‐κB pathway (Figure 3D). As expected, inhibition of ROS levels with NAC or alpinetin suppressed the nuclear translocation of p65/NF‐κB (Figure 3E).

### 3.4 Alpinetin dampens the expression of HIF‐1α by ROS/ NF‐κB axis

**FIGURE 4:**
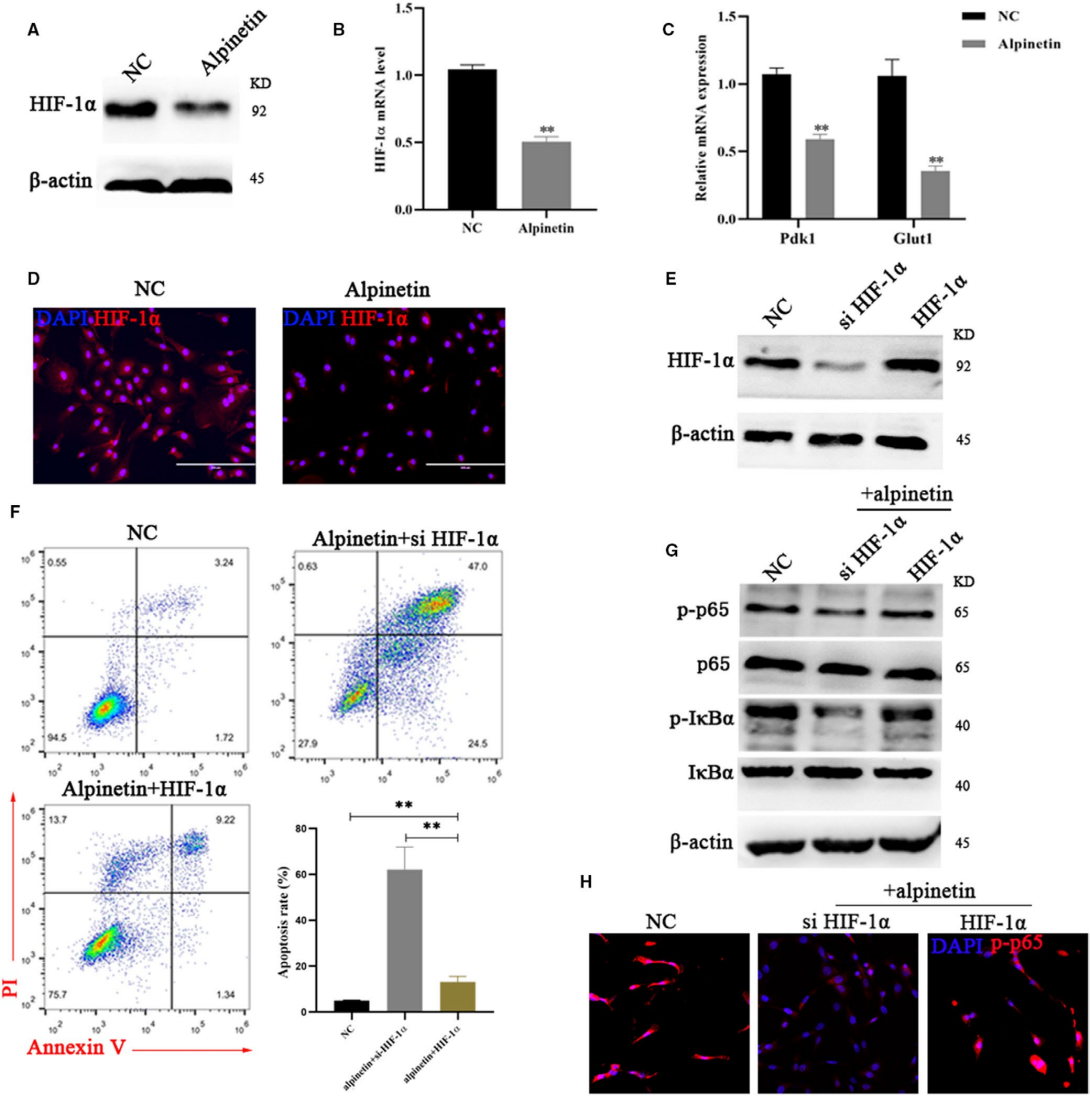
Alpinetin dampens the expression of HIF‐1α. A, Western blot analysis of HIF‐1α protein levels under alpinetin treatment, β‐Actin was used as an internal control. B, qPCR analysis of HIF‐1α gene expression from total mRNA isolated from breast cancer cells within alpinetin. C, qPCR analysis of Pdk1and Glut1 gene expression from total mRNA isolated from breast cancer cells within alpinetin. D, Immunofluorescence staining HIF‐1α of in 4T1 cells treated with alpinetin. DAPI (blue), HIF‐1α (red), Scale bar: 100 μm. E, Western blot analysis of HIF‐1α protein levels under si‐ HIF‐1α or pcDNA3.1 + HIF‐1α transfected. β‐Actin was used as an internal control. F, Cell apoptosis assays of 4T1 cells treated with NC, alpinetin (50 μmol/L) in presence si‐HIF‐1α or pcDNA3.1 + HIF‐1α using FACS. Cells were collected and labelled with Annexin V‐FITC and PI. G, Western blot analyses of p65, p‐p65, IκBα and p‐IκBα after treatment with the above regimens in 4T1 cells. β‐Actin was used as an internal control. H, Analysis of p65 nuclear after treatment with the above regimens in 4T1 cells. NF‐kB was detected by immunostaining with antibodies against p‐p65 (red) and DAPI (blue). Bars, 50 mm. All results are expressed as the mean ± SEM of three independent experiments. The symbols * and ** denote significant differences of *P* < .05 and *P* < .01, respectively

Next, we look for functional effectors downstream of NF‐κB to understand the molecular mechanisms by which alpinetin exert their synergistic effects on breast cancer cell lines. We found that HIF‐1α levels were significantly lower in alpinetin treatment cells (Figure 4A,B), which are a central regulator of a global metabolic transcription programme and participate in the development of breast cancer. Furthermore, we assessed the expression of established target genes of HIF‐1a, including phosphoinositide‐dependent protein kinase‐1 (Pdk1) and glucose transporter type 1 (Glut1) and found that their expression was similarly dampened in alpinetin treatment group (Figure 4C) and IF experiment results are as expected (Figure 4D). The reduction of key proteins in the glycolytic pathway such as GLUT1 indicates that the cancer cells have insufficient energy supply, which in turn induces apoptosis or inhibition migration. The flow cytometry analyses showed that in alpinetin treatment cells, overexpression of HIF‐1α abolishes it induced apoptosis (Figure 4E,F). Interestingly, overexpression of HIF‐1α activated NF‐κB signalling pathway, which indicates that the presence of NF‐κB/HIF‐1α positive feedback pathway allows low doses of alpinetin to play a greater role (Figure 4G,H).

### 3.5 Alpinetin inhibits tumour growth in vivo

**FIGURE 5:**
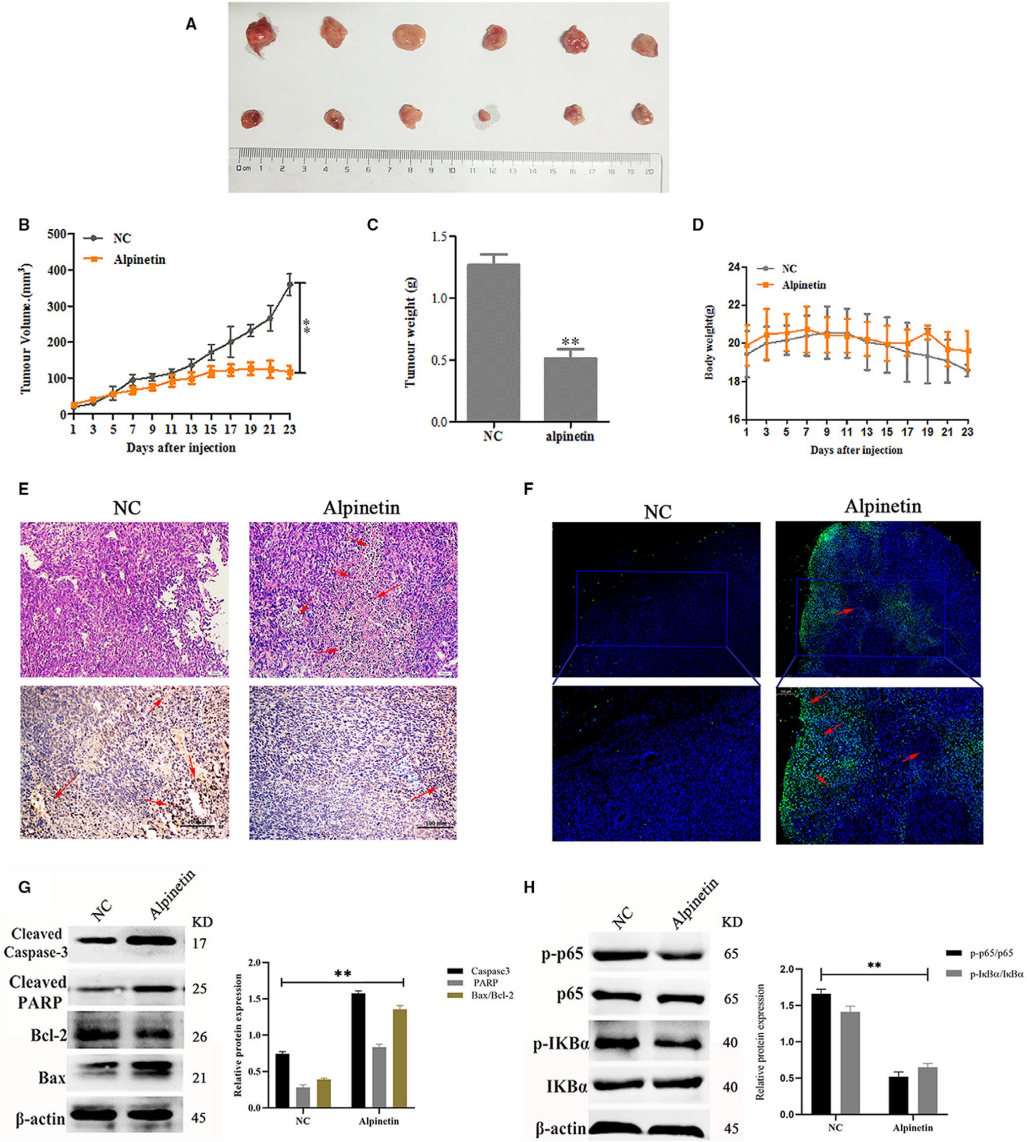
Alpinetin inhibits tumour growth in vivo*.* BALB/c female mice were divided into two groups. After 8 d, the indicated drug was injected, and the tumour volume and mouse body weight were measured every 2 d. On day 23, the mice were sacrificed, and samples were collected. A‐D, Morphological assessment of tumour volume (A and B), tumour weight (C) and mouse body weight (D). E, Representative panels of H&E staining; red arrows indicate the main necrotic cells; scale bar, 50 μm. Representative immunohistochemical staining for Ki67 in tumour tissue is also shown; red arrows indicate positive cells; scale bar, 200 μm. F, Apoptosis in tumour sections was examined by TUNEL staining. Blue spots represent cell nuclei, and green spots represent TUNEL‐positive cells. G and H, The protein expression levels of p65, p‐p65, IκBα, p‐IκBα, PARP, caspase 3, Bcl‐2 and Bax were detected by Western blot. β‐Actin was used as an internal control. All results are expressed as the mean ± SEM of three independent experiments. The symbol **denotes significant differences of *P* < .01

Finally, the antitumour effect of alpinetin in vivo was evaluated with 4T1 tumour‐bearing BALB/c mice. Morphological studies showed that the tumour volume and tumour weight in the control group clearly increased compared with those in the alpinetin treatment group (Figure 5A‐C). However, there was no significant change in body weight, and this result implies a low toxicity of alpinetin in vivo (Figure 5D). Furthermore, no evident histopathological abnormalities were observed in the vital organs, such as the heart, liver and kidney, by H&E staining (data not shown). However, we found significant tumor cell apoptosis in the alpinetin‐treated group (Figure 5E). In addition, alpinetin showed efficient antiproliferative activity and induced apoptosis in tumour tissue as assessed by immunohistochemistry, TUNEL assay and Western blot (Figure 5E‐H). We also examined the expression of key apoptotic proteins and proteins in the NF‐κB signalling pathway. Similar to results seen in in vitro experiments, alpinetin was shown to promote apoptosis by inhibiting the activation of the NF‐κB signalling pathway (Figure 5H). Taken together, these observations indicate that alpinetin exerts superior anticancer activity both in vitro and in vivo.

## 4 DISCUSSION

**FIGURE 6:**
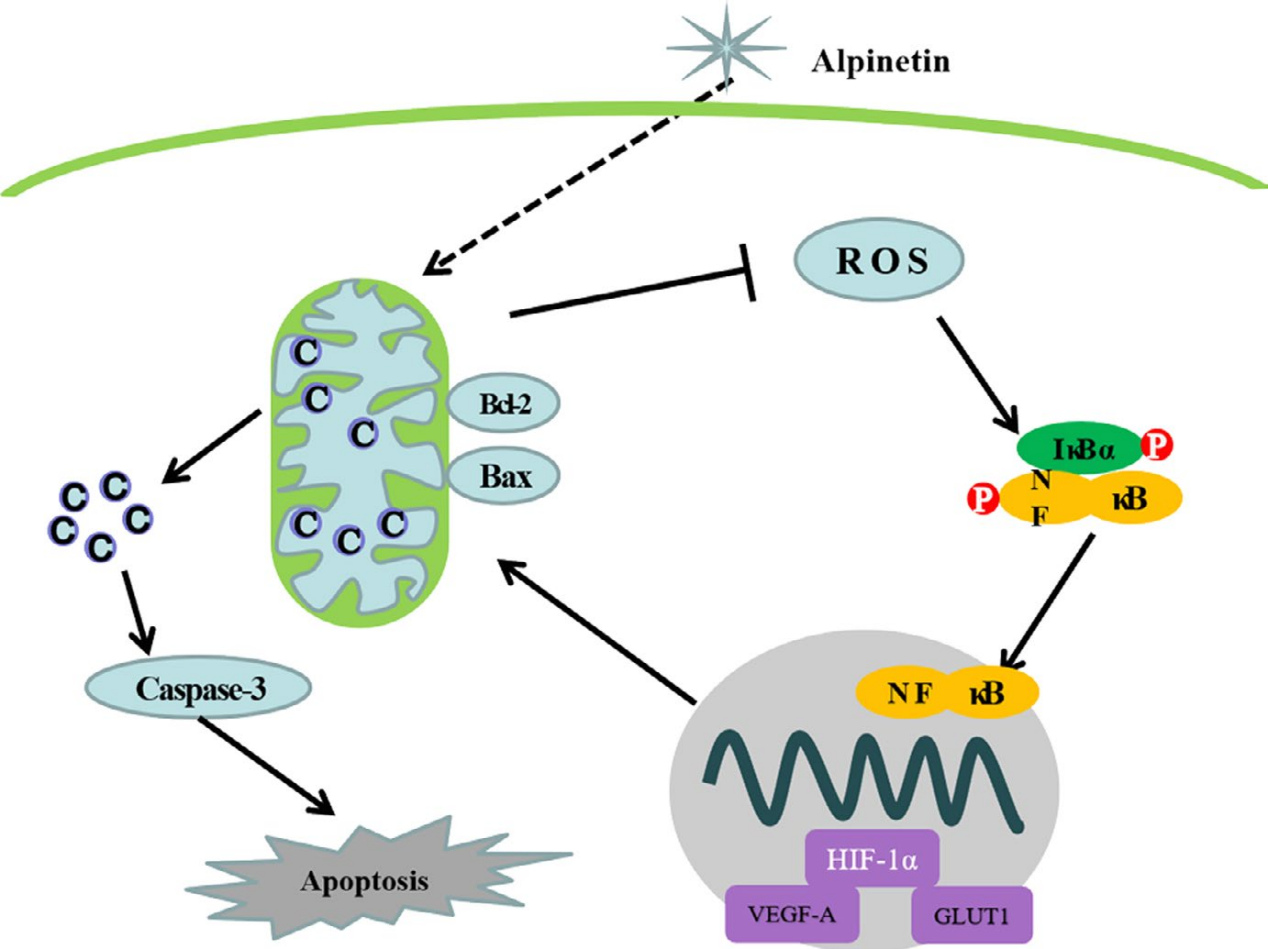
Schematic illustration delineating the role of alpinetin therapy. Alpinetin induced release of cytochrome c and then mitochondria‐dependent apoptosis in breast cancer cells. Mitochondrial dysfunction leads to reduced intracellular ROS generation and then inhibits activated NF‐κB pathway. Defective NF‐kB‐mediated HIF‐1α gene inhibits cancer cell proliferation and migration via its hundreds of oncogenic target genes

Worldwide, there were approximately 2.1 million newly diagnosed female breast cancer cases in 2018, accounting for almost 1 in 4 cancer cases amongst women.^1^ Finding effective drugs for fighting breast cancer has always been a major goal of scientists. This current study is a comprehensive report of a major mechanism of action by which alpinetin alleviates tumour burden. The results show that alpinetin reduced ROS production, resulting in reduced ROS/NF‐Κb/HIF‐1α pathway activity, which leads to impaired cell proliferation and induction of mitochondria‐associated apoptosis, culminating in reduced tumour growth (Figure 6).

Despite previous reports showing that alpinetin induces apoptosis in mouse models of various cancers and that alpinetin exhibits anti‐inflammatory activity,^24^, ^25^, ^26^ the underlying mechanisms remain largely unclear. Here, we showed that alpinetin inhibits proliferation and induces apoptosis in a dose‐dependent manner in breast cancer (Figure 1). The integrity of mitochondrial function is critical for influencing the intrinsic apoptotic pathway.^27^ The changes of mitochondrial membrane potential (Δψm) and release of cytochrome c both indicate dysfunction of mitochondria (Figure 2). As mitochondria are the vital of cellular energy supply and the hubs for signal transmission, dysfunction of mitochondria was frequently linked to human disorders, such as cancer and Barth syndrome. The integrity of mitochondria directly affects the balance of ROS in cells. In cancer, ROS generally accepted is tumour suppressors that are participated in tumorigenesis, progression and survival phenotypes.^23^, ^28^ However, the effect of ROS within cancer cells depending on several factors, like stimulus and cell type, even stimulates duration, this is a double‐edged sword. In this study, we found that alpinetin efficiently decreases intracellular ROS induced mitochondria‐associated apoptosis in breast cancer.

This is not surprising, and we hypothesized that alpinetin can regulate a variety of cancer apoptotic pathways, such as the NF‐κB pathway, which are amongst ROS‐activating transcription factors.^19^ Further studies have shown that alpinetin can inhibit the phosphorylation of p‐p65 and interfere with its entry into the nucleus (Figure 3). The NF‐κB pathway exerts survival activity by inducing the expression of several anti‐apoptotic genes, such as c‐FLIP, XIAP and member of the Bcl‐2 family.^29^, ^30^ It is worth noting that activation of the NF‐κB signalling pathway led to increased DNA damage in inflammatory disease,^31^, ^32^ but it promotes cancer cell survival in many types of cancer.^33^ The reason is that the effects of NF‐κB depend on the stimulus factors and cell type, and it can suppress or induce autophagy in a context‐dependent manner.^34^

In the present study, inhibition of HIF‐1α‐mediated apoptosis and migratory characteristics of breast cancer cells, through inactivated of NF‐κB pathway, was also confirmed (Figure 4). HIF‐1α is the master transcriptional regulator mediating the adaptive responses to intra‐tumoral hypoxia to drive cancer progression, particularly in breast cancer, which the efficiency of cellular oxygen utilization is lower than normal cells in its microenvironment.^15^ HIF‐1α transcriptionally regulates hundreds of oncogenic genes that are involved in cell fate, angiogenesis, invasion, metastasis and metabolic adaptation, such as VEGF, CXCR4 and LOX.^17^ knockdown of HIF‐1α leads to reduced migration and invasion of various breast cancer and HIF‐1α also vital maintenance survival and self‐renewal in tumour stem cells and metastasis cancer cell.^35^, ^36^ We showed noted that alpinetin decreases the expression of HIF‐1α on transcription level by inactivating NF‐κB pathway. However, there are other ways to affect the stability or degradation of HIF‐1α, such as ubiquitination and proteasomal degradation, which needs further research to prove. Importantly, overexpression HIF‐1α dose not to completely abrogate alpinetin‐induced apoptosis (Figure 4F), these findings indicated that other pathways may also contribute to alpinetin‐induced cancer phenotypic changes.

In conclusion, we report the discovery of a traditional Chinese medicine, alpinetin, that induces mitochondria‐associated apoptosis and suppresses proliferation in a dose‐dependent manner. The pharmacological effects of alpinetin were also confirmed in an MDA‐MB‐231 mouse xenograft model. Mechanistically, our study provides proof that alpinetin exerts an antitumour effect through the ROS/NF‐κB/HIF‐1α axis. Collectively, these observations suggest that alpinetin could act as a potential breast cancer chemotherapy agent.

## CONFLICT OF INTEREST

The authors declare no competing financial interests.

## AUTHORS’ CONTRIBUTIONS

TZ, SG, GD and CQ conceived and designed the experiments. TZ, XZ, GD and CQ performed the experiments. TZ, SG, XZ, JQ and CQ analysed the data. TZ and GD wrote the paper. All authors read and approved the final manuscript.

## Data Availability

The data that support the findings of this study are available from the corresponding author upon reasonable request.
